# Investigation on the role of nsSNPs in HNPCC genes – a bioinformatics approach

**DOI:** 10.1186/1423-0127-16-42

**Published:** 2009-04-24

**Authors:** C George Priya Doss, Rao Sethumadhavan

**Affiliations:** 1 Bioinformatics Division, School of Biotechnology, Chemical and Biomedical Engineering, Vellore Institute of Technology, Vellore 632014, Tamil Nadu, India

## Abstract

**Background:**

A central focus of cancer genetics is the study of mutations that are causally implicated in tumorigenesis. The identification of such causal mutations not only provides insight into cancer biology but also presents anticancer therapeutic targets and diagnostic markers. Missense mutations are nucleotide substitutions that change an amino acid in a protein, the deleterious effects of these mutations are commonly attributed to their impact on primary amino acid sequence and protein structure.

**Methods:**

The method to identify functional SNPs from a pool, containing both functional and neutral SNPs is challenging by experimental protocols. To explore possible relationships between genetic mutation and phenotypic variation, we employed different bioinformatics algorithms like Sorting Intolerant from Tolerant (SIFT), Polymorphism Phenotyping (PolyPhen), and PupaSuite to predict the impact of these amino acid substitutions on protein activity of mismatch repair (MMR) genes causing hereditary nonpolyposis colorectal cancer (HNPCC).

**Results:**

SIFT classified 22 of 125 variants (18%) as 'Intolerant." PolyPhen classified 40 of 125 amino acid substitutions (32%) as "Probably or possibly damaging". The PupaSuite predicted the phenotypic effect of SNPs on the structure and function of the affected protein. Based on the PolyPhen scores and availability of three-dimensional structures, structure analysis was carried out with the major mutations that occurred in the native protein coded by *MSH2 and MSH6 *genes. The amino acid residues in the native and mutant model protein were further analyzed for solvent accessibility and secondary structure to check the stability of the proteins.

**Conclusion:**

Based on this approach, we have shown that four nsSNPs, which were predicted to have functional consequences (*MSH2*-Y43C, *MSH6*-Y538S, *MSH6*-S580L, *and MSH6*-K854M), were already found to be associated with cancer risk. Our study demonstrates the presence of other deleterious mutations and also endorses with *in vivo *experimental studies.

## Background

Colorectal cancer is the second leading cause of cancer death in the western countries after lung cancer. Colorectal cancer manifests itself after an accumulation of several genetic alterations. These mutations can be either somatic or inherited. The most common forms of heritable colorectal cancer are hereditary non-polyposis colorectal cancer (HNPCC) and familial adenomatous polyposis (FAP). Hereditary nonpolyposis colorectal cancer is an autosomal dominant syndrome which accounts for about 1–5% of colorectal cancer [1]. Lynch syndrome, also known as hereditary nonpolyposis colorectal cancer (MIM# 114500) is the most common form of inherited colorectal cancer caused by mutations in high-penetrance genes. Hereditary nonpolyposis colorectal cancer is a dominant condition, meaning that people with HNPCC have a 50% chance of passing the HNPCC gene mutation (change) to each of their children. With HNPCC, the lifetime risk for colorectal cancer (CRC) is approximately 80% and the lifetime risk of endometrial cancer is 40%. HNPCC is associated with germline genetic alterations in the mismatch repair (MMR) genes. The primary function of the mismatch repair system is to eliminate single base substitutions and insertion-deletion errors that may arise during DNA replication. The system involves several proteins encoded by 5 different genes namely [*MLH1 *(MIM# 120436), *MSH2 *(MIM# 609309), *MSH6 (*MIM# 600678), *PMS1 *(MIM# 600258), and *PMS2 *(MIM# 600259)] have been implicated in HNPCC [2]. Loss of mismatch repair gene activity leads to an accumulation of replication errors and genetic instability that is exhibited as micro satellite instability (MSI). Germline mutations in *MLH1 *and *MSH2 *account for approximately 90% of detected mutations in families with HNPCC where as mutations in *MSH6 *account for about 7%–10%, and *PMS2 *mutations in fewer than 5% of families with Hereditary nonpolyposis colorectal cancer and risk of developing colorectal cancer is also increased among *MSH2 *mutation carriers as compared with *MLH1 *mutation carriers [3].

In human genome more than 99% genetic nucleotides are same, only less than 1% genetic variations are different. These genetic variations widely spread on species genome which form a ubiquitous phenomenon cause the differences and diversities of the species. The variation in DNA may consist of deletions where some pieces are missing, insertions of new genetic material or changes in nucleotides, where a sequence is changed to another. Most of the variation in human genome consists of substitutions in single nucleotide, where one of the four nucleotides (A, T, G, and C) has changed to another one. The phenomenon of having such a varying nucleotide at a certain locus is referred as single nucleotide polymorphism (SNP). Common definition of the SNP requires that the relative frequency of the least frequent allele is greater than 0.01. Single nucleotide polymorphisms are generally the most common form polymorphisms of DNA sequence variation in the species genome and resource for mapping complex genetic traits. There are now several databases with these variations of single nucleotide polymorphisms, such as the human genome variation database, HGVBase [4] and the National Center for Biotechnology Information (NCBI) database, dbSNP [5]. With exception of variants lying in promoters or splice site donors or acceptors, it is difficult to determine the effect of non-coding SNPs on gene expression. For this reason, particular attention has been focused towards nonsynonymous coding SNPs (nsSNPs), SNPs that cause amino acid alteration. These types of alterations are believed to be more likely to cause a change in structure and as such compromise the function of a protein. Our literature survey shows that nsSNPs affect the functional roles of proteins in signal transduction of visual, hormonal and other stimulants [6,7] in gene regulation by altering DNA and transcription factor binding [8,9]. nsSNPs may inactivate functional sites of enzymes or alter splice sites and thereby form defective gene products [10,11]. They may destabilize proteins, or reduce protein solubility [12], may have functional effects on transcriptional regulation, by affecting transcription factor binding sites in promoter or intronic enhancer regions [13], or alternatively splicing regulation by disrupting exonic splicing enhancers or silencers [14]. To understand the mechanism of phenotypic variations due to nsSNPs, it is important to assess the structural consequences of the alteration of amino acid residue. With the advent of high-throughput SNP detection techniques, the number of known nsSNPs is growing rapidly, providing an important source of information for studying the relationship between genotypes and phenotypes of human diseases.

**Figure 1:**
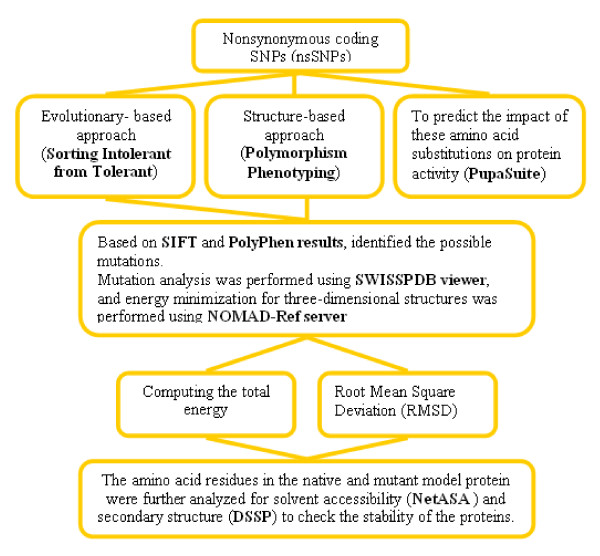
**Proposed methodology for the functional nonsynonymous coding SNPs analysis**.

Over the past few years, quite a lot of studies have attempted to predict the functional consequences of an nsSNPs whether it is disease-related or neutral, based on sequence information and structural attributes [15] using computational algorithms such as SIFT and PolyPhen algorithms to screen for deleterious nsSNPs [16,17]. The structure of a protein can change in various ways due to the biochemical differences of the amino acid variant (acidic, basic, or hydrophobic) and by the location of the variant in the protein sequence (by affecting tertiary or quaternary structure or the active site where substrate binds) which can have a deleterious effect on the structure and/or function of the proteins [18]. Therefore, it is important to determine whether an nsSNP that affects the amino acid sequence of a gene product can alter protein function and contribute to disease will be a challenge in the coming years [19]. Several groups have tried to evaluate the deleterious nsSNPs based on 3-dimensional (3D) structure information of proteins by *in-silico *analysis. They indicated that the residue solvent accessibility, which could identify the buried residues, was confidently proposed as predictors of deleterious substitutions [20,21]. Deleterious nsSNPs analyses for the HNPCC genes have not been estimated computationally until now, although they have been the focus for experimental researchers. Therefore, in this work, the computational algorithms namely SIFT, PolyPhen, PupaSuite, ASA View and DSSP were used to identify the deleterious nsSNPs that are likely to affect the function and structure of the protein. Based on PolyPhen, we identified the possible mutation, proposed a model structure for the mutant proteins and compared this with the native protein in the 3-D modeled structure of the *MSH2 *and *MSH6 *gene. We further analyzed native and mutant modeled proteins for solvent accessibility and secondary structure analysis. Secondary structures and solvent accessibilities of amino acid residues give a useful insight into the structure and function of a protein [22-25]. We have described our approach using computational tools to provide related information of SNPs and a guide to experimental biologists (Figure 1). Our computational study also demonstrates the presence of other deleterious mutations in other HNPCC genes in which there is no availability of three- dimensional structure that may affect the expression and function of proteins with possible roles in colon cancer.

## Materials and methods

### Database Mining for SNPs

The list of HNPCC genes studied were obtained from the Online Mendelian Inheritance in Man (OMIM) . We used Human genome variation database, HGVBase  and National Center for Biotechnology Information (NCBI) database dbSNP  for the retrieval of SNPs and their related protein sequence of five genes namely *MLH1*, *MSH2*, *MSH6*, *PMS1 *and *PMS2 *causing HNPCC for our computational analysis.

### Evaluation of coding single nucleotide polymorphisms

There are many web-based resources available that allow one to predict whether nonsynonymous coding SNPs may have functional effects on proteins. We chose two complementary algorithms for functional impact prediction of nsSNPs: Sorting Intolerant From Tolerant (SIFT)  and Polymorphism Phenotyping (PolyPhen) [26,27]. Protein conservation analysis was performed using the SIFT developed by Ng and Henikoff. SIFT (Sorts Intolerant From Tolerant) version 2.0 was used to distinction between functional and non-functional coding mutations and predicts whether an amino acid substitution in a protein will have a phenotypic effect. SIFT is based on the premise that protein evolution is correlated with protein function. Variants that occur at conserved alignment positions are expected to be tolerated less than those that occur at diverse positions. The algorithm uses a modified version of PSIBLAST [28] and Dirichlet mixture regularization [29] to construct a multiple sequence alignment of proteins that can be globally aligned to the query sequence and belong to the same clade. The underlying principle of this program is that it generates alignments with a large number of homologous sequences and assigns scores to each residue, ranging from zero to one. SIFT scores ≤ 0.05 are predicted by the algorithm to be intolerant or deleterious amino acid substitutions, whereas scores >0.05 are considered tolerant [30]. Higher the tolerance index of a particular amino acid substitution, lesser is its likely impact.

PolyPhen is a computational tool for identification of potentially functional nsSNPs. Predictions are based on a combination of phylogenetic, structural and sequence annotation information characterizing a substitution and its position in the protein. For a given amino acid variation, PolyPhen performs several steps: (a) extraction of sequence-based features of the substitution site from the UniProt database, (b) calculation of profile scores for two amino acid variants, (c) calculation of structural parameters and contacts of a substituted residue. PolyPhen scores were classified as 'benign', 'possibly damaging', or 'probably damaging' [16]. PolyPhen searches for three-dimensional protein structures, multiple alignments of homologous sequences and amino acid contact information in several protein structure databases. Then, it calculates position-specific independent counts (PSIC) scores for each of two variants, and computes the difference of the PSIC scores of the two variants. The higher a PSIC score difference, the higher functional impact a particular amino acid substitution is likely to have. A PSIC score difference of 1.5 and above is considered to be damaging.

### Analyzing the molecular phenotypic effects of SNPs

PupaSuite are now synchronized to deliver annotations for both non-coding and coding SNP, as well as annotations for the SwissProt set of human disease mutations. It is an integrated interface of PupaSNP [31] and PupasView [32] for selecting SNPs with potential phenotypic effect accessible via  and through . In this approach, the input consists of a list of genes (genes belonging to a given pathway, involved in a particular biological function, etc.) and the user must specify the type of gene identifiers by selecting either Ensembl or an external database (which include GenBank, Swissprot/TrEMBL and other gene ids supported by Ensembl). PupasView retrieves SNPs that could affect conserved regions that the cellular machinery uses for the correct processing of genes (intron/exon boundaries or exonic splicing enhancers). It uses algorithms like Tango (β-aggregation regions in protein sequences) and FoldX (stability change caused by the single amino acid variation) to predict the effect of coding non-synonymous SNPs on several phenotypic properties such as structure and dynamics, functional sites and cellular processing of human proteins using either sequence-based or structural bioinformatics tools and additional methods for predicting SNPs in TFBSs and splice sites [33].

### Modeling nsSNP locations on protein structure and their RMSD difference

Structural analyses were performed based on the crystal structure of the protein for evaluating the structural stability of native and mutant protein. We used the web resource SAAPdb [34] and dbSNP to identify the protein coded by *MSH2 *and *MSH6 *gene (PDB ID 2O8C). We also confirmed the mutation positions and the mutation residues from this server. These mutation positions and residues were in complete agreement with the results obtained with SIFT and PolyPhen programs. The mutation was performed using SWISSPDB viewer, and energy minimization for 3D structures was performed using NOMAD-Ref server [35]. This server use Gromacs as default force field for energy minimization based on the methods of steepest descent, conjugate gradient and L-BFGS methods [36]. We used the conjugate gradient method for optimizing the three dimensional structures. We computed the energy gives the information about the protein structure stability and Root Mean Square Deviation (RMSD) criteria provide widespread understanding of deviation at structure level. Deviation between the two structures was evaluated by their RMSD values.

### Analyzing the effects of mutations on protein stability based on solvent accessibility and secondary structure analysis

Solvent accessibility is the ratio between the solvent accessible surface area of a residue in a three dimensional structure and in an extended tripeptide conformation. We obtained the solvent accessibility information using NetASAView [37]. The entire implementation of ASAView for all PDB proteins, as a whole or for an individual chain may be accessed at . Requirements for the use are simply the PDB code or the coordinate file. Solvent accessibility was divided into three classes, buried, partially buried and exposed indicating, respectively, low, moderate and high accessibility of the amino acid residues to the solvent [38,39]. For a successful analysis of the relation between amino acid sequence and protein structure, an unambiguous and physically meaningful definition of secondary structure is essential. We obtained the information about secondary structures of the proteins using the program DSSP [40]. The prediction of solvent accessibility and secondary structure has been studied as an intermediate step for predicting the tertiary structure of proteins.

## Results

### SNP dataset

Five genes namely *MLH1*, *MSH2*, *MSH6*, *PMS1 *and *PMS2 *with a potential role for the cause of HNPCC were retrieved from Online Mendelian Inheritance in Man. For our investigations, we selected SNPs in (i) non-synonymous coding regions, (ii) 5' and 3' UTR regions, and (iii) intronic regions. Out of 1970 SNPs, 125 were non-synonymous SNPs (nsSNPs) and 68 SNPs in coding synonymous region. Non-coding region is comprised of 44 SNPs in UTR and 1733 were in the intronic region.

### Predictions of deleterious and damaging coding nsSNPs

Protein conservation analysis was performed using the SIFT algorithm predicts whether an amino acid substitution may have an impact on protein function by aligning similar proteins, and calculating a score which is used to determine the evolutionary conservation status of the amino acid of interest. One twenty five nsSNPs retrieved from six genes were submitted independently to the SIFT program to check its tolerance index. SIFT scores [16] were classified as intolerant (0.00–0.05), potentially intolerant (0.051–0.10), borderline (0.101–0.20), or tolerant (0.201–1.00). The higher the tolerance index, the less functional impact a particular amino acid substitution is likely to have, and vice versa. It can be seen from (Table 1) that six percent of the nsSNPs exhibit SIFT scores of 0.0, eleven percent of the variants have scores between 0.01–0.05 and three percent of the variants have scores between 0.006–0.10 respectively. Thus eighteen percent nsSNPS are classified as 'intolerant' showed a highly deleterious tolerance index score of 0.00–0.05 and could affect the protein function in the HNPCC genes.

**Table 1:** nsSNPS that were predicted to be functionally significant by SIFT (Tolerance index) and PolyPhen (PSIC score).

					**SIFT**		**PolyPhen**	
					
**Gene ids**	**SNP ids**	**Alleles**	**Amino acid change**	**Reference**	**Tolerance index**	**Predicted impact**	**PSIC score**	**Predicted impact**
*MLH1*	rs41295280	C/G	G22A	[45,46]	**0.03**	Intolerant	**1.606**	Possibly damaging
	rs11541859	C/G	E89Q	-NA-	0.04	Intolerant	1.012	Borderline
	rs41295282	A/G	S93G	[46-48]	0.07	Potentially Intolerant	1.828	Possibly damaging
	rs35338630	C/G	R264G	[46,49]	**0.00**	Intolerant	**1.711**	Possibly damaging
	rs41295284	A/T	L607H	[45,46,50]	0.06	Potentially Intolerant	1.665	Possibly damaging
	rs35045067	A/G	Y646C	[46]	**0.00**	Intolerant	**2.978**	Probably damaging
	rs2020873	C/T	H718Y	[51,52]	0.09	Potentially Intolerant	2.738	Probably damaging
*MSH2*	rs17217723	A/G	Y43C	[17,46]	**0.00**	Intolerant	**2.970**	Probably damaging
	rs33946261	C/G	H46Q	[45,46,53]	0.25	Tolerant	2.988	Probably damaging
	rs17217772	A/G	N127S	[17,46,54]	**0.01**	Intolerant	**2.359**	Probably damaging
	rs4987188	A/G	G322D	[17,46,55]	0.37	Tolerant	1.504	Possibly damaging
	rs17224367	C/T	L390F	-NA-	0.02	Intolerant	0.949	Benign
	rs35717997	C/T	P415S	[46]	**0.05**	Intolerant	**1.982**	Possibly damaging
	rs180522	T/G	H639Q	[46,56]	**0.00**	Intolerant	**3.352**	Probably damaging
	rs41295290	A/G	D646G	-NA-	**0.05**	Intolerant	**2.410**	Probably damaging
	rs41294982	C/T	P670L	[46,56]	**0.00**	Intolerant	**3.379**	Probably damaging
	rs34319539	A/T	K909I	-NA-	**0.05**	Intolerant	**1.863**	Possibly damaging
	rs41295182	G/T	L911R	[46,56]	**0.04**	Intolerant	**1.961**	Possibly damaging
*MSH6*	rs41294988	A/C	K13T	[45]	**0.01**	Intolerant	**1.722**	Possibly damaging
	rs1042821	C/T	G39E	[57,58]	0.82	Tolerant	1.530	Possibly damaging
	rs41294984	C/T	S65L	[45]	0.25	Tolerant	1.620	Possibly damaging
	rs3211299	A/C	S144I	[45]	**0.02**	Intolerant	**1.883**	Possibly damaging
	rs41295268	A/G	R468H	[45]	0.54	Tolerant	1.954	Possibly damaging
	rs728619	A/C	Y538S	-NA-	0.78	Tolerant	2.674	Probably damaging
	rs41295270	C/T	S580L	[45]	0.19	Borderline	2.399	Probably damaging
	rs35552856	A/C	K728T	[59]	0.41	Tolerant	1.539	Possibly damaging
	rs34374438	A/T	K854M	[58,60]	**0.04**	Intolerant	**2.087**	Probably damaging
	rs2020912	C/T	V878A	[57,58]	0.52	Tolerant	1.540	Possibly damaging
	rs41295278	A/G	R1321G	[45]	0.07	Potentially Intolerant	1.975	Possibly damaging
*PMS1*	rs5742973	C/G	E27Q	-NA-	**0.03**	Intolerant	**1.507**	Possibly damaging
	rs1145231	C/T	M394T	[61]	0.63	Tolerant	1.950	Possibly damaging
	rs55726197	C/G	Q437H	-NA-	0.18	Borderline	2.057	Probably damaging
	rs56305733	A/G	Q450R	-NA-	0.59	Tolerant	1.655	Possibly damaging
	rs1145232	A/G	G501R	[61]	0.49	Tolerant	2.367	Probably damaging
	rs2066456	A/G	N632S	-NA-	0.74	Tolerant	1.961	Possibly damaging
	rs56309301	A/C	N855T	-NA-	0.53	Tolerant	1.722	Possibly damaging
*PMS2*	rs56203955	G/T	Q30P	[61]	**0.00**	Intolerant	**2.838**	Probably damaging
	rs6977072	C/G	P37A	-NA-	**0.02**	Intolerant	**1.503**	Possibly damaging
	rs34506829	A/G	E41K	-NA-	**0.00**	Intolerant	**2.052**	Probably damaging
	rs35943120	A/T	L42I	-NA-	**0.00**	Intolerant	**1.547**	Possibly damaging
	rs35629870	A/G	R151H	-NA-	**0.04**	Intolerant	**2.292**	Probably damaging
	rs36038802	A/C	Q160K	-NA-	0.69	Tolerant	1.544	Possibly damaging

NA-Not Available; nsSNPs which were found to be deleterious by both SIFT and PolyPhen were highlighted as bold.

The structural levels of alteration were determined by applying the PolyPhen program. It predicts the functional effect of amino acid changes by considering evolutionary conservation, the physiochemical differences, and the proximity of the substitution to predicted functional domains and/or structural features. All the 125 nsSNPs from 5 genes submitted to SIFT were also submitted as input to the PolyPhen server. Table 1 presents the distribution of the variants by PolyPhen score. Note that the directionalities of the SIFT and PolyPhen scores are opposite and the SIFT scores are limited to the range of 0.0 to 1.0, while the PolyPhen scores in this dataset ranged from 3.37 to 0.0. PolyPhen scores of >2.0, scores expected to be "Probably damaging" to protein structure and function [41], account for thirteen percent of the nsSNPs and nineteen percent of the nsSNPs exhibited PolyPhen scores of 1.99-1.50, scores indicative of variants that are "Possibly damaging" to protein function. Amino acid variants can impact the folding, interaction sites, solubility or stability of proteins. To understand the relationship between genetic and phenotypic variation, it is essential to assess the structural consequences of the respective non-synonymous mutations in proteins. To identify how often a disease phenotype can be explained by a destructive effect on protein structures or functions, we have mapped known disease mutations onto known three-dimensional structures of proteins based on PolyPhen score. The nsSNPs with ids namely rs17217723, rs180522 and rs41294982 showed a PSIC score difference ≥ 2.9 at positions Y43C, H639Q and P670L in *MSH2 *gene while the nsSNPs with ids namely rs728619, rs41295270 and rs34374438 showed a PSIC score difference ≥ 2.0 at positions Y538S, S580L and K854M in *MSH6 *gene were selected for modeling analysis based on the availability of the 3D structure. To date, data on the validity of these algorithms has come from benchmarking studies based on the analysis of "known" deleterious substitutions annotated in databases, such as Swiss-Prot, shown to successfully predict the effect of over 80% of amino acid substitutions [16,41-43]. Experimental studies of individual proteins have also confirmed the accuracy of SIFT and PolyPhen [16,44]. Hence, we could infer that the results obtained by the evolutionary-based approach (SIFT) correlated well with the results obtained by structural-based approach (PolyPhen), as can be seen from (Table 1). The nsSNPs which were predicted to be deleterious in causing an effect in the structure and function of the protein by SIFT and PolyPhen correlated well experimental studies [45-61] as shown in (Table 1).

### Predictions of potential phenotypic effect in SNPs

The effect of non-synonymous coding SNPs can be analyzed by means of the physico-chemical properties of the affected proteins. PupaSuite tries to pinpoint the exact effect of a mutation to a specific structural or physico-chemical property, ranging from protein aggregation to the disruption of protein-protein interactions, or from changes in protein turnover rate to sub-cellular (mis) localisation. *In-silico *methods provide a useful tool for an initial approach to any mutation suspected of causing aberrant RNA processing. These mutations can result either in complete skipping of the exon, retention of the intron or in the introduction of a new splice site within an exon or intron. In rare cases, mutations that do not disrupt or create a splice site, activate preexisting pseudo splice sites consistent with the proposal that introns contain splicing inhibitory sequences [62]. Nonsense and missense mutations can disrupt exonic splicing enhancers (ESEs) and cause the splicing machinery to skip the mutant exon, with dramatic effects on the structure of the gene product [63]. ESEs are common in alternative and constitutive exons, where they act as binding sites for Ser/Arg-rich proteins (SR proteins), a family of conserved splicing factors that participate in multiple steps of the splicing pathway [64]. Out of 54 SNPs reported in (Table 2), 45 nsSNPs disrupted the exonic splicing enhancers, 3 nsSNPs disrupted the exonic splicing silencers, 3 SNPs in mRNA disrupted the exonic splicing silencers, 1 SNP in mRNA disrupted the exonic splicing enhancers and 1 SNP in intron region involved in intron/exon junctions. Evidence in support of varied levels of alternative splicing is available for some Lynch syndrome related mutations [65,66]. It is a noteworthy finding in our computational approach that 19 nsSNPs with ids namely (rs11541859, rs35045067, rs17217723, rs33946261, rs4987188, rs17224367, rs35717997, rs34319539, rs1042821, rs2020912, rs3211299, rs35552856, rs728619, rs1145231, rs1145232, rs2066456, rs35629870, rs35943120, rs36038802) disrupted the exonic splicing enhancers were also found to be damaging by SIFT and PolyPhen analysis. Our methodology can be used to prioritize SNPs that might play important role for large epidemiologic studies and genetic analysis.

**Table 2:** List of SNPs in HNPCC genes predicted by PupaSuite.

**Gene ids**	**SNP ids**	**Region**	**Functional significance**
*MLH1*	rs11541859	Coding nonsynonymous	Exonic splicing enhancers
	rs1799977	Coding nonsynonymous	Exonic splicing enhancers
	**rs1800149**	Coding nonsynonymous	Exonic splicing enhancers
	rs34213726	Coding nonsynonymous	Exonic splicing enhancers
	rs34285587	Coding nonsynonymous	Exonic splicing enhancers
	rs35045067	Coding nonsynonymous	Exonic splicing enhancers
	rs35831931	Coding nonsynonymous	Exonic splicing enhancers
	rs1803985	mRNA	Exonic splicing silencers
*MSH2*	rs17217716	Coding nonsynonymous	Exonic splicing enhancers
	rs17217723	Coding nonsynonymous	Exonic splicing enhancers
	rs17224367	Coding nonsynonymous	Exonic splicing enhancers
	rs1802577	Coding nonsynonymous	Exonic splicing enhancers
	rs33946261	Coding nonsynonymous	Exonic splicing enhancers
	rs34136999	Coding nonsynonymous	Exonic splicing enhancers
	rs34319539	Coding nonsynonymous	Exonic splicing enhancers
	rs34986638	Coding nonsynonymous	Exonic splicing enhancers
	rs35107951	Coding nonsynonymous	Exonic splicing enhancers
	rs35717997	Coding nonsynonymous	Exonic splicing enhancers
	rs35784190	Coding nonsynonymous	Exonic splicing enhancers
	rs4987188	Coding nonsynonymous	Exonic splicing enhancers
	rs12476364	intron	intron/exon junctions
*MSH6*	rs1042821	Coding nonsynonymous	Exonic splicing enhancers
	rs2020908	Coding nonsynonymous	Exonic splicing enhancers
	rs2020912	Coding nonsynonymous	Exonic splicing enhancers
	rs3136334	Coding nonsynonymous	Exonic splicing enhancers
	rs3211299	Coding nonsynonymous	Exonic splicing enhancers
	rs34014629	Coding nonsynonymous	Exonic splicing enhancers
	rs35462442	Coding nonsynonymous	Exonic splicing enhancers
	rs35552856	Coding nonsynonymous	Exonic splicing enhancers
	rs35946687	Coding nonsynonymous	Exonic splicing enhancers
	rs728619	Coding nonsynonymous	Exonic splicing enhancers
	rs3211299	Coding nonsynonymous	Exonic splicing silencers
	rs34938432	Coding nonsynonymous	Exonic splicing silencers
*PMS1*	rs1145231	Coding nonsynonymous	Exonic splicing enhancers
	rs1145232	Coding nonsynonymous	Exonic splicing enhancers
	rs1145234	Coding nonsynonymous	Exonic splicing enhancers
	rs2066456	Coding nonsynonymous	Exonic splicing enhancers
	rs2066459	Coding nonsynonymous	Exonic splicing enhancers
	rs5742932	mRNA	Exonic splicing enhancers
	rs5742932	mRNA	Exonic splicing silencers
	rs5742933	mRNA	Exonic splicing silencers
*PMS2*	rs10254120	Coding nonsynonymous	Exonic splicing enhancers
	rs1805318	Coding nonsynonymous	Exonic splicing enhancers
	rs1805321	Coding nonsynonymous	Exonic splicing enhancers
	rs1805322	Coding nonsynonymous	Exonic splicing enhancers
	rs1805323	Coding nonsynonymous	Exonic splicing enhancers
	rs2228007	Coding nonsynonymous	Exonic splicing enhancers
	rs35629870	Coding nonsynonymous	Exonic splicing enhancers
	rs35690297	Coding nonsynonymous	Exonic splicing enhancers
	rs35911407	Coding nonsynonymous	Exonic splicing enhancers
	rs35943120	Coding nonsynonymous	Exonic splicing enhancers
	rs36038802	Coding nonsynonymous	Exonic splicing enhancers
	rs35943120	Coding nonsynonymous	Exonic splicing silencers

### Modeling and analysis of mutant structure

**Figure 2:**
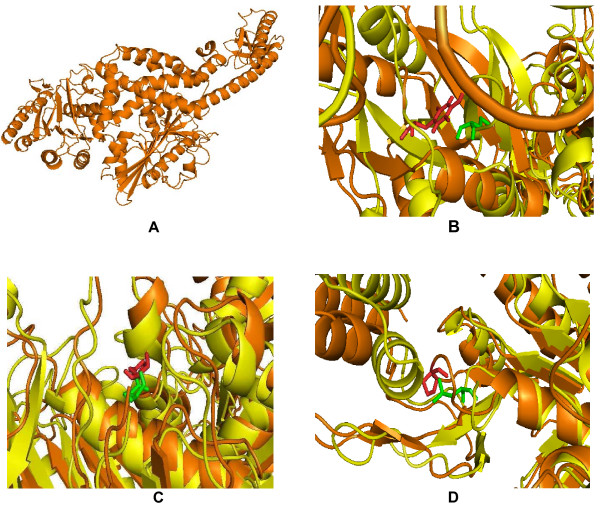
**(A) Native structure of MSH2 gene with 'A' chain of PDB ID **2O8C**(orange)**. (B) Superimposed structure of native tyrosine (orange) with mutant amino acid cysteine (pale green) at 43 position in 2O8C with RMSD 4.30 Å. (C) Superimposed structure of native histidine (orange) with mutant amino acid glutamine (pale green) at 639 position in 2O8C with RMSD 3.93 Å. (D) Superimposed structure of native proline (orange) with mutant amino acid leucine (pale green) at 670 position in 2O8C with RMSD 3.65 Å.

**Figure 3:**
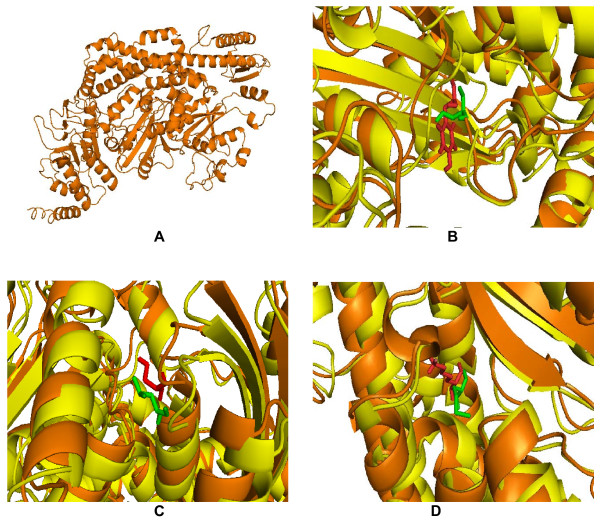
**(A) Native structure of MSH6 gene with 'B' chain of PDB ID **2O8C**(orange)**. (B) Superimposed structure of native tyrosine (orange) with mutant amino acid serine (pale green) at 538 position in 2O8C with RMSD 3.52 Å. (C) Superimposed structure of native serine (orange) with mutant amino acid leucine (pale green) at 580 position in 2O8C with RMSD 3.37 Å. (D)Superimposed structure of native lysine (orange) with mutant amino acid methionine (pale green) at 854 position in 2O8C with RMSD 3.30 Å.

Single amino acid mutations can significantly change the stability of a protein structure. So, the knowledge of a protein's three-dimensional (3D) structure is essential for a full understanding of its functionality. Mapping the deleterious nsSNPs into protein structure information was obtained from dbSNP and SAAPdb. The available structure for the *MSH2 and MSH6 *gene is reported to have a PDB ID (2O8C). Mutation analysis was performed based on the results obtained from highest PolyPhen scores. The mutations for 2O8C at their corresponding positions were performed by SWISS-PDB viewer independently to achieve modeled structures. Then, energy minimizations were performed by NOMAD-Ref server for the native type protein 2O8C and the mutant type structures. It can be inferred from (Table 1) that nsSNPs in *MSH2 *gene with ids namely rs17217723, rs180522 and rs41294982 showed the highest PolyPhen scores 2.970, 3.352 and 3.379 respectively. According to this, the mutation occurred for native protein in the 'A' chain of PDB ID 2O8C at position Y43C with an SNP ID (rs17217723), H639Q with an SNP ID (rs180522) and P670L with an SNP ID (rs41294982) based on PolyPhen results. It can be seen that the total energy for mutant type structure Y43C, H639Q and P670L were found to be -53305.15, -53377.01, -53405.59 Kcal/mol respectively. The RMSD values between the native type (2O8C) and the mutant Y43C is 4.30 Å, between native type and the mutant H639Q is 3.93 Å and between native type and the mutant P670L is 3.65 Å. The total energy and RMSD value of mutant structure Y43C is high when compared to the other mutants H639Q and P670L respectively. Similarly, for *MSH6 *gene based on the PolyPhen scores, mutation analysis was performed in nsSNPs with IDs namely rs728619, rs41295270 and rs34374438 respectively. According to this, the mutation occurred for native protein in the 'B' chain of PDB ID 2O8C at position Y538S with an SNP ID (rs728619), S580L with an SNP ID (rs41295270) and K854M with an SNP ID (rs34374438). It can be seen that the total energy for mutant type structure Y538S, S580L and K854M were found to be -58509.39, -58513.55, -58506.94 Kcal/mol respectively. The RMSD values between the native type (2O8C) and the mutant Y538S is 3.52 Å, between native type and the mutant S580L is 3.37 Å and between native type and the mutant K854M is 3.30 Å. The total energy and RMSD value of mutant structure Y43C is high when compared to the other mutants H639Q and P670L in *MSH2 *gene, while all the three mutants Y538S, S580L and K854M in *MSH6 *showed almost same total energy and RMSD. Higher the RMSD value more will be the deviation between native and mutant type structures and which in turn changes their functional activity. The superimposed structures of the native protein 2O8C (chain A) with the three mutant type proteins Y43C, H639Q and P670L of MSH2 gene are shown in shown in (Figure 2a, b, c &2d) and the superimposed structures of the native protein 2O8C (chain B) with the three mutant type proteins Y538S, S580L and K854M of *MSH6 *gene are shown in (Figure 3a, b, c &3d) respectively.

Based on the SIFT, PolyPhen, total energy and RMSD values of the mutant proteins, solvent accessibility and secondary structure of all the residues in the native protein and mutant protein Y43C, H639Q and P670L of *MSH2 *gene and Y538S, S580L and K854M of *MSH6 *gene were computed with NetASA. Solvent accessibilities and secondary structures of amino acid residues give a useful insight into the structure and function of a protein [22-24]. In the folded structure of a protein polar and charged side chains have higher solvent accessibility than non-polar side chains, suggesting that formation of a hydrophobic core is a strong driving force in protein folding [67]. The prediction of residue solvent accessibility can help in better understanding the relationship between sequence and structure. The residues namely Glu(16), Met(26), Val(63), Ile(224), Asn(263), Ala(370), Arg(373), Pro(385), Ala(398), Pro(415), Pro(591), Ile(624) and Cys(822) showed a change in solvent accessibility from an buried to exposed state in the mutant protein Y43C and Leu(11), Gly(18), Phe(23), Lys(29), Thr(32), Tyr(43), Glu(86), Asp(91), Asn (105), Asn(115), Lys(122), Leu(128), Ser(129), Gln(130), Asp(133), Asn(138), Met(152), Ser(153), Ala(154), Tyr(165), Ser(168), Arg(171), Lys(172), Glu(177), Gln(183), Ile(194), Pro(202), Asp(209), Arg(214), Arg(219), Gly(220), Ile(224), Tyr(238), Gln(239), Asn(242), Gly(247), Glu(251), Ala(256), Glu(258), Glu(278), Asp(282), Gln (288), Leu(291), Tyr(299), Gly(315), Gln(344), Trp(345), Lys(347), Arg(389), Gln(395), Tyr(405), Glu(422), Ser(448), Glu(455), Asp(459), Pro(472), Ser(479), Met(485), Ser(498), Asp(502), Leu(505), Asp(514), Thr(526), Asn(535), Asp(597), Val(598), Leu(625), Val(644), Phe(634), Gln(662), Lys(675), Thr(677), Arg(680), Ser(699), Gly(712), Ala(727), Ser(738), Glu(786), Leu(811), Val(817) and Ala(843) showed a change in solvent accessibility from an exposed to buried state in the mutant protein Y43C. It is interesting to note that mutant position Y43C, itself changed the solvent accessibility from exposed to buried state. The mutant amino acid cysteine is hydrophobic in nature. Most of the information in the solvent-accessibility features comes from the fact that buried residue positions are most likely to be adversely effected by amino-acid substitutions, due to loss of structural stability [[68,69], and [41]]. Many studies have suggested that hydrophobic core residues are likely sites of deleterious mutations. Hence, change in solvent accessibility from an exposed to buried state could be considered functionally significant in the mutant protein at structural level [21]. The occurrence of weak interactions has been observed at the terminus of the secondary structural units, in particular a-helix and β-sheets [70,71]. These interactions play a definitive role in stabilizing these structures of proteins. The propensity of the amino acid residues to favor a particular conformation has been well documented. Such conformational preference is not dependent on the amino acid alone but is also dependent on the local amino acid sequence. We analyzed the secondary structure of each amino acid residue in the native and mutant structures of the protein. We found that the residues namely Asp(133), Ile (134), Leu(135), Arg(219), Gly(219), Ile(237), Tyr(238), Gln(252), Met(253), Asn (254), Ser(255), Ala(256), Val(257), Pro(259), Glu(260), Met(261), Glu(262), Glu(368), Asp(369), Arg(396), Gln(413), Glu(422), Lys(423), Phe(447), Ala(640), Cys(641), Val (642), Glu(643), Arg(737), Ser(738) and Glu(853) changed their conformation from turn in the native protein to helix conformation in the mutant protein, Gln(239), Asp(240), Leu(241), Lys(430), Leu(431), Leu(432), Leu(433), Ala(434), Val(435) and Phe(436) changed from bend to helix, Ile(304), Leu(330), Thr(457) and Thr(772) changed from helix to turn and Leu(279), Leu(280), Ser(281), His(785), Glu(786), Leu(787), Thr(788) changed their conformation from bend to turn in the mutant protein. The results of solvent accessibility and secondary structure analysis for the rest of the mutations H639Q and P670L of *MSH2 *gene and Y538S, S580L and K854M of *MSH6 *gene are provided in Additional file 1. Therefore, understanding the functional consequences of non-synonymous changes and predicting the potential causes and the molecular basis of diseases involves integration of information from multiple heterogeneous sources including sequences, structure data, solvent accessibility and secondary structure analysis.

## Discussion

A major interest in human genetics is to distinguish mutations that are functionally neutral from those that contribute to disease. Amino acid substitutions currently account for approximately half of the known gene lesions responsible for human inherited disease [72]. Therefore, the identification of nsSNPs that affect protein functions and relate to disease is an important task. The effect of many nsSNPs will probably be neutral as natural selection will have removed mutations on essential positions. Assessment of non-neutral SNPs is mainly based on phylogenetic information (i.e. correlation with residue conservation) extended to a certain degree with structural approaches (PolyPhen). However, there is increasing evidence that many human disease genes are the result of exonic or noncoding mutations affecting regulatory regions [73,74]. Much attention has been focused on modeling by different methods the possible phenotypic effect of SNPs that cause amino acid changes, and only recently has interest focused on functional SNPs affecting regulatory regions or the splicing process. Moreover, because of their widespread distribution on the species genome, SNPs become particularly important and valuable as genetic makers in the research for the diseases and corresponding drug. Currently, millions of human SNPs have reported by high-throughput methods. The vast number of SNPs causes a challenge for biologists and bioinformaticians although they provide lot information about the relationships between individuals. Besides numerous ongoing efforts to identify millions of these SNPs, there is now also a focus on studying associations between disease risk and these genetic variations using a molecular epidemiological approach. This plethora of SNPs points out a major difficulty faced by scientists in planning costly population-based genotyping, which is to choose target SNPs that are most likely to affect phenotypic functions and ultimately contribute to disease development.

Currently, most molecular studies are focusing on SNPs located in coding and regulatory regions, yet many of these studies have been unable to detect significant associations between SNPs and disease susceptibility. To develop a coherent approach for prioritizing SNP selection for genotyping in molecular studies, we applied an evolutionary perspective to SNP screening. We correlated findings from molecular studies of cancer with the evolutionary conservation levels of non-synonymous SNPs using a sequence homology-based tool. Our hypothesis was that, amino acids conserved across species are more likely to be functionally significant. Therefore, SNPs that change these amino acids might be more likely to be associated with cancer susceptibility. It is becoming clear that application of the molecular evolutionary approach may be a powerful tool for prioritizing SNPs to be genotyped in future molecular epidemiological studies. Therefore, our analysis will provide useful information in selecting SNPs that are likely to have potential functional impact and ultimately contribute to an individual's cancer susceptibility.

Out of 1970 SNPs, 125 were non-synonymous SNPs (nsSNPs) of the HNPCC genes were submitted to the SIFT and PolyPhen algorithms. Sorting Intolerant from Tolerant (SIFT) classified 22 of 125 variants (18%) as "Intolerant." Polymorphism Phenotyping (PolyPhen) classed 40 of 125 amino acid substitutions (32%) as "probably or possibly damaging". 49 nsSNPs, 3 SNPs in mRNA and a SNP in intron region showed molecular phenotypic variation by PupaSuite. Based on the PolyPhen scores and availability of 3D structures, structure analysis was carried out with the major mutation that occurred in the native protein coded by *MSH2 and MSH6 *genes. The total energy and RMSD value of mutant structure Y43C is high when compared to the other mutants H639Q and P670L in *MSH2 *gene, while all the three mutants Y538S, S580L and K854M in *MSH6 *showed almost same total energy and RMSD. Based on the SIFT, PolyPhen, total energy and RMSD values of the mutant proteins, solvent accessibility and secondary structure of all the residues in the native protein and mutant protein Y43C, H639Q and P670L of *MSH2 *gene and Y538S, S580L and K854M of *MSH6 *gene were computed with NetASA. Solvent accessibilities and secondary structures of amino acid residues give a useful insight into the structure and function of a protein. Based on this approach, we have shown that four nsSNPs, which were predicted to have functional consequences (*MSH2*-Y43C, *MSH6*- Y538S, *MSH6*- S580L, *and MSH6*- K854M), were already found to be associated with cancer risk.

## Conclusion

Our current analysis focuses on SNPs in the coding regions, and our findings could explain a significant fraction of the cancer risk that has been detected. This approach might also be applied to a relationship between SNP conservation levels and epidemiological studies of diseases other than cancer. More importantly, this study builds a bridge from evolutionary biology to molecular epidemiology, which may further our understanding of disease-related SNPs and ultimately facilitate SNP genotyping in future studies. In summary, we have systematically and comprehensively evaluated structure and sequence-based computational prediction methods applied to variants in the *MLH1*, *MSH2*, *MSH6*, *PMS2 *and *PMS1 *genes and provided detailed structural explanations for the measured and predicted impact of *MSH2 *and *MSH6 *variants. The data presented here show that this novel bioinformatics approach to classifying cancer-associated variants is robust and can be used for large-scale analyses. Our approach will present the application of computational tools in understanding functional variation from the perspective of structure, expression, evolution and Phenotype. The existing *in silico *methods that we used can also be adapted by any investigator to a priori SNP selection or post hoc evaluation of variants identified in whole-genome scans. The best-supervised learning algorithms are in greater agreement with experimental results than has been reported previously.

## Abbreviations

CRC: Colorectal cancer; ESEs: Exon splicing enhancers; HNPCC: Hereditary non-polyposis colorectal cancer; HGVBase: Human genome variation database; FAP: Familial adenomatous polyposis; MMR: Mismatch repair; NCBI: National Center for Biotechnology Information; NsSNPs: Nonsynonymous single nucleotide polymorphism; OMIM: Online Mendelian Inheritance In Man; MSI: Micro satellite instability; PSIC: Position-specific independent counts; RMSD: Root Mean Square Deviation; SNP: Single Nucleotide Polymorphism; SIFT: Sorting Intolerant From Tolerant.

## Competing interests

The authors declare that they have no competing interests.

## Authors' contributions

CGPD carried out the SNP analysis in the HNPCC genes. CGPD collected the SNP data from the databases, analyzed the SNPs using different algorithms and predicted the deleterious SNPs. RS carried out the modeling analysis and drafted the manuscript. All authors read and approved the final manuscript.

## Supplementary Material

Additional File 1 **Tables S1 and S2**. The results of solvent accessibility and secondary structure analysis for the rest of the mutations H639Q and P670L of *MSH2 *gene and Y538S, S580L and K854M of *MSH6 *gene are provided in Tables S1& S2. Table S1: Solvent accessibility in the native and mutant proteins. Table S2: Secondary structure analysis in the native and mutant proteins. Click here for file
