# Supplementary material for: Investigation on the role of nsSNPs in HNPCC genes – a bioinformatics approach
Source: J Biomed Sci. 2009 Apr 24;16(1):42. doi: 10.1186/1423-0127-16-42 (PMC2682794; doi:10.1186/1423-0127-16-42)
Supplement: Additional File 1 — Tables S1 and S2. The results of solvent accessibility and secondary structure analysis for the rest of the mutations H639Q and P670L of MSH2 gene and Y538S, S580L and K854M of MSH6 gene are provided in Tables S1& S2. Table S1: Solvent accessibility in the native and mutant proteins. Table S2: Secondary structure analysis in the native and mutant proteins. [file 1423-0127-16-42-S1.doc]

**Supplementary Table 1**

Solvent accessibility in the native and mutant proteins.

| **Mutation**  **Position** | **Changed from exposed to buried** | **Changed from buried to exposed** |
| --- | --- | --- |
| H639Q | Gln(4), Leu(11), Gly(18), Phe(23), Arg(39), Asp(91), Asn(115), Leu(128), Ser(129), Glu(132), Asp(133), Ile(145),Lys(151), Met (152), Tyr(165), Ser(168), Arg(171), Glu(177), Gln(183), Pro(202), Asp(209), Arg( 214), Arg(219), Gly(220), Ser(233), Gln(239), Asn(242), Gly(247), Glu(251), Glu(260), Gln (288), Leu(291), Thr(292), Asp(295), Tyr(299), Arg(308),Asn(311), Gly(315),Ser(316), Ser(323), Arg(340), Trp(345), Lys(347), Arg(389), Lys(393), Gln(395), Tyr(405), Ser(448), Glu(455), Asp(459), Pro(472), Ser(479), Met(485), Ser (498), Asp(502), Leu(505), Asp(514), Thr(526), Asn(535), Asp(597), Val(598), Asn(613), Ala(615), Leu (625), Ile(648), Phe(650), Gln(662), Lys(675),Ser(676), Thr(677), Arg(680), Ser(699), Val(712), Gly(713), Ala(727), Leu(730), Ala(733), Ser(738), Glu(749), Phe(760), Leu(811), Ile(828), Ala(844) | Glu(16), Arg(99), Glu(101), Leu(119), Ala(120), Lys(122), Asp(167), Ile(224), Leu(341), Leu(350), Ala(370), Arg(373), Ala(398) Pro(415), Ile(624), Cys(822) |
| P670L | Leu(11), Gly(18), Phe(23), Lys(29), Arg(39), Tyr(43), Asp(91), Asn(115), Leu(128), Ser(129), Met(152), Ser(153), Ala(154), Tyr(165), Gln(183), Ile(194), Asp(209), Met(210), Arg( 214), Arg(219), Gly(220), Ser(233), Gln(239), Asn(242), Gly(247), Glu(260), Asn(263), Glu(278), Leu(280), Gln (288), Leu(291), Thr(292), Tyr(299), Gly(315), Ser(323), Leu(341), Arg(389), Lys(392), Gln(395), Tyr(405), Leu(432), Ser(448), Glu(455), Asp(459), Pro(472), Ser(479), Met(485), Ser (498), Asp(502), Leu(505), Thr(526), Ile(577), Asp(597), Val(598), Leu (625), Phe(650), Gln(662), Lys(675), Ser(676), Leu(687), Gln(690), Ser(699), Val(712), Ala(727), Leu(730), Ser(738), Ile(794), Thr(810), Gly(827), Val(830), Ala(844) | Glu(16), Ala(120), Lys(122), Asp(167), Ile(224), Asn(263), Ala(370), Pro(415), Pro(591), Ile(624) |
| Y538S | Glu(368), Glu(381), Asp(390), Met(410), Arg(411), Phe(421), Ile(442), Asn(455), Tyr(469),Asp(471), Ser(472), Thr(488), Val(508), Glu(512), Ser(525), Val(526), Lys(537), His(552), Lys (567), Gln(572), Pro(590), Lys(598), Ser(631), Lys(638), Glu(645), Asp(649), Gly(650), Val(653), Ser(666), Asp(667), Leu(671), Lys(693), Asn(705), Leu(712), Asp(715), Arg(732), Asn(741), Gly(756), Gln(776), Asp(793), Glu(796), Lys(804), Glu(819),Lys(833), Ser(834), Ser(840), Ile(843), Glu(847), Glu(876), Leu(893), Asn(897), Glu(908), Phe(916), Ser(950), Gly(963), Tyr(969), Gly(973), Tyr(977), Glu(980), Thr(986), Asn(989), Glu(1012), Lys(1014), Ala(1016), Asn(1020), Glu(1023), Asp(1026), Leu(1029), Lys(1030), Asp(1031), Arg(1034), Tyr(1038), Tyr(1044), Asp(1046), Gln(1048), Gly(1072), Met(1074), Arg(1095), Cys(1098), Glu(1118), Asn(1136), Ser(1141(, Thr(1142), Ala(1206), Thr(1225), His(1248), Tyr(1249), Ser(1252), Ala(1261), Arg(1263), Leu(1264), Thr(1284), Lys(1296), Ser(1297), Tyr91298), Asn(1301), Asn(1302), Ile(1313), Gly(1316), Ala(1320), Glu(1324), Asn(1327), Ser(1329), Arg(1331) | Thr(672), Pro(673), Asn(742) Thr(764) Val(801), Ile(874), Arg(901), Gln(939), Glu(946), Lys(1013), His(1096), Thr(1219), Lys(1240), His(1266), Ala(1268), Leu(1332), Phe(1333) |
| S580L | Arg(384), Asp(390), Ala(393), Met(410), Lys(412), Phe(421), Asp(422), Ile(442),Gly(454), Trp(456), Tyr(469), Asp(471), Ser(472), Gln(475), Thr(486), Thr(488), Ser(525), Val(526), Pro(531), Asn(534), Lys(537), His(552), Thr(553), Lys (567), Gln(572), Pro(590), Lys(598), Glu(604), Ser(631), Lys(638), Glu(645), Asp(649), Gly(650), Val(653), Ser(668), Gly(674), Asn(705), Leu(712), Asp(715), Arg(732), Asn(742), Arg(761), Asp(763), Gln(776), Asn(789), Asp(793), Glu(796), Val(800), Val(801), Lys(804), Glu(819), Lys(824), Val(828), Leu(832), Lys(833), Ser(834), Glu(847), Glu(876), Leu(893), Asn(897), Glu(908), His(918), Gly(963), Tyr(969), Gly(973), Tyr(977), Glu(980), Thr(986), Glu(1012), Lys(1014), Ala(1016), Asn(1020), Glu(1023), Asp(1026), Leu(1029), Lys(1030), Asp(1031), Arg(1034), Tyr(1038), Tyr(1044), Asp(1046), Gln(1048), Gly(1069), Gly(1072), Met(1074), Glu(1118), Ser(1141), Thr(1142), Ala(1206), Asn(1229), His(1248), Tyr(1249), Asp(1255), Ser(1257), Ala(1261), Arg(1263), Leu(1264), Thr(1284), Ser(1297), Tyr(1298), Asn(1301), Asn(1302), Ala(1320), Glu(1324), Asn(1327), Ser(1329), Arg(1331) | Glu(512), Arg(581), Asn(742), Asp(763), Leu(832), Ile(874), Arg(901), Gln(939), Glu(946), His(1096), Leu(1159), Thr(1219), His(1266), Ala(1268) |
| K854M | Glu(368), Glu(381), Gly(409), Met(410), Arg(411), Phe(421), Ile(442), Val(444), Gly(454), Tyr(469), Asp(471), Ser(472), Thr(488), Glu(490), Tyr(505), Val(508), Ser(525), Val(526), His(552), Thr(553), Lys (567), Gln(572), Glu(604), Ser(631), Lys(638), Glu(645), Val(653), Ser(666), Leu(671), Asn(705), Leu(712), Asp(715), Arg(732), Gly(756), Gln(776), Asn(789), Asp(793), Glu(796), Lys(804), Glu(819), Lys(824), Val(828), Lys(833), Ser(834), Glu(847), Ser(860), Glu(876), Leu(893), Asn(897), Phe(916), Ser(950), Gly(963), Tyr(969), Gly(973), Tyr(977), Glu(980), Thr(986), Asn(989), Glu(1012), Lys(1014), Ala(1016), Asn(1020), Glu(1023), Asp(1031), Arg(1034), Tyr(1038), Tyr(1044), Asp(1046), Gln(1048), Gly(1072), Met(1074), Arg(1095), Glu(1118), Ser(1141), (Thr1142), Ala(1206), Thr(1225), Asn(1229), His(1248), Tyr(1249), Ser(1251), Asp(1255), Ser(1255), Ala(1261), Arg(1263), Leu(1264), Ser(1297), Tyr(1298), Asn(1301), Asn(1302), Ala(1320), Glu(1324), Asn(1327), Ser(1329) | Glu(512), Tyr(535), Arg(581), Leu(617), Gly(620), Thr(672), Asn(742), Asp(763), Val(801), Ile(874), Arg(901), Gln(939), His(1096), Leu(1159), Thr(1219), His(1266), Ala(1268) |

**Supplementary Table 2**

Secondary structure analysis in the native and mutant proteins.

| **Mutation**  **Position** | **Secondary Structure analysis** |
| --- | --- |
| H639Q | **T→H**: Gly(25), Arg(219), Ile(237), Gln(252), Met(253), Asn(254), Ser(255), Ala(256), Val(257), Glu(368), Asp(369), Gln(377), Arg(396), Gln(413), Glu(422), Lys(423), Phe(436), Phe(447), Glu(464), Ser(586), Gly(587), Tyr(588), Val(589), Ala(640), Cys(641), Val(642), Glu(643), Gly(674), Gln(690), Ile(691), Phe(725), Glu(853)  **H→T**: Leu(401), Thr(772)  **3→H**: Tyr(238), Gln(239), Asp(240), Leu(241), Lys(430), Leu(431), Leu(432), Leu(433), Ala(434), Val(435)  **3→T**:Leu(279), Leu(280)  **T→3**:Glu(132), Asp(133), His(785), Glu(786), Leu(787), Thr(788)  **T→S**: Cys(697), Glu(698) |
| P670L | **T→H**: Gly(25), Ile(294), Arg(219), Ile(237), Gln(252), Met(253), Asn(254), Ser(255), Ala(256), Glu(368), Asp(369), Gln(377), Arg(396), Gln(413), Glu(422), Lys(423), Phe(447), Glu(464), Ser(586), Gly(587), Tyr(588), Val(589), Ala(640), Cys(641), Val(642), Glu(643), Gly(674), Gln(690), Ile(691), Phe(725), Ala(789), Glu(853)  **H→T**: Ile(304), Leu(330), Leu(401), Thr(772)  **3→H**: Tyr(238), Gln(239), Asp(240), Leu(241), Lys(430), Leu(431), Leu(432), Leu(433), Ala(434), Val(435)  **3→T**: Leu(279), Leu(280)  **T→3**:Glu(132), Asp(133), Pro(259), Glu(260), Met(261), Glu(262), Lys(380), His(785), Glu(786), Leu(787), Thr(788)  **T→S**: Lys(197), Cys(697), Glu(698) |
| Y538S | **T→H**: Ser(405), Lys(498), Lys(610), Ser(702), Leu(712), Asp(713), Ser(714), Asp(715), Thr(716), Lys(813), Lys(814), Thr(914), Ala(915), Ile(962), Gly(963), Ser(1067), Glu(1322), Phe(1323), Lys(1325), Met(1326), Asn(1327), Gln(1328), Ser(1329), Leu(1330), Arg(1331), Leu(1332), Phe(1333)  **H→T**: Gln(419), Gln(835), Asn(836), Asp(936)  **3→H**: Tyr(366), His(367), Glu(368), Thr(369), Pro(838), Asp(839), Ser(840), Arg(841), Arg(959), Asn(960), Arg(961)  **3→T**: Glu(371), Trp(372), Leu(373), Glu(463), Ile(464), Ala(465), Arg(507), Glu(675), Lys(676), Ser(677)  **T→3**: Thr(563), Ser(564), Leu(565), Tyr(642), Phe(643), Arg(644), Glu(983), Asn(984), Phe(985)  **T→S**: Ala(1162), Glu(1163)  **S→T**: Phe(1285) |
| S580L | **T→H**: Asn(404), Ser(405), Lys(498), Lys(610), Leu(712), Asp(713), Ser(714), Asp(715), Thr(716), Val(800), Lys(813), Lys(814), Thr(914), Ala(915), Ile(962), Gly(963), Ser(1067), Met(1156), Glu(1322), Phe(1323), Lys(1325), Met(1326), Asn(1327), Gln(1328), Ser(1329), Leu(1330), Arg(1331), Leu(1332), Phe(1333)  **H→T**: His(437), Asp(936)  **3→H**: Tyr(366), His(367), Glu(368), Thr(369), Pro(838), Asp(839), Ser(840), Arg(841), Arg(959), Asn(960), Arg(961)  **3→T**: Glu(371), Trp(372), Leu(373)  **T→3**: Tyr(642), Phe(643), Arg(644), Glu(983), Asn(984), Phe(985)  **T→S**: Ala(1162), Glu(1163)  **S→T**: Phe(1285) |
| K854M | **T→H**: Asn(404), Ser(405), Lys(498), Ser(702), Leu(712), Asp(713), Ser(714), Asp(715), Thr(716), Lys(813), Lys(814), Thr(914), Ala(915), Ile(962), Gly(963), Ser(1067), Glu(1322), Phe(1323), Lys(1325), Met(1326), Asn(1327), Gln(1328), Ser(1329), Leu(1330), Arg(1331), Leu(1332), Phe(1333)  **H→T**: Asp(936)  **3→H**: Tyr(366), His(367), Glu(368), Thr(369), Pro(838), Asp(839), Ser(840), Arg(841), Arg(959), Asn(960), Arg(961)  **3→T**: Glu(371), Trp(372), Leu(373), Glu(675), Lys(676), Ser(677)  **T→3**: Tyr(642), Phe(643), Arg(644), Glu(983), Asn(984), Phe(985)  **T→S**: Thr(563), Ser(564), Leu(565), Ala(1162), Glu(1163)  **S→T**: Phe(1285 |
